# Supplementary material for: Practices of Self-Care in Healthy Old Age: A Field Study
Source: Geriatrics (Basel). 2023 May 13;8(3):54. doi: 10.3390/geriatrics8030054 (PMC10204451; doi:10.3390/geriatrics8030054)
Supplement: Supplementary file 1 [file geriatrics-08-00054-s001.zip › geriatrics-2310780-supplementary.pdf]

Supplementary Materials

Table S1. Difference between sociodemographic variables and survival self-care practices.

	**IADL**							**BADL**						
	**Frequency**				**Variety**			**Frequency**				**Variety**		
	N	∑	SD	t	∑	SD	t	N	∑	SD	t	∑	SD	t
Participants	101	3.55	1.33		3.20	1.04		105	2.56	0.48		4.03	0.66	
**Age**														
<75	51	3.54	1.33	-.260	3.25	1.04	.548	55	2.45	0.38	-2.693*	3.93	.67	-1.666
>75	50	3.55	1.35		3.14	1.07		50	3.08	0.47		4.14	.64	
**Sex**														
Male	13	2.41	1.46	3.355**	2.43	1.02	3.084**	17	2.47	0.48	.951	3.94	.66	.597
Female	88	4.06	1.26		3.32	1.00		88	2.58	0.43		4.05	.66	
**Education level**														
Basic	55	4.02	1.34	.921	3.29	1.15	.973	57	3.00	0.46	.996	4.11	.62	1.304
Advanced	46	3.45	1.33		3.09	.91		48	2.52	0.45		3.94	.71	
**Convivence**														
Alone	45	3.38	1.23	-1.524	3.20	1.01	.017	46	2.53	0.48	-.561	4.09	.63	.808
Accompanied	56	4.07	1.40		3.19	1.09		59	2.58	0.41		3.98	.68	
**Confident**														
Yes	55	4.05	1.45	1.171	3.31	1.02	1.183	56	2.47	0.37	-2.371*	4.02	.75	-.187
No	46	3.43	1.17		3.07	1.08		49	3.07	0.50		4.04	.55	

*p< .05; **p<.005

Note: basic education = 8 or fewer years of schooling; advanced education =9 or more years of schooling.

Table S2. Difference between sociodemographic variables and maintenance self-care practices.

	Physical							Cognitive							Social							Spiritual						
		Frequency			Variety				Frequency			Variety				Frequency			Variety				Frequency			Variety		
	N	∑	SD	t	∑	SD	t	N	∑	SD	t	∑	SD	t	N	∑	SD	t	∑	SD	t	N	∑	SD	t	∑	SD	t
Total	98	2.43	1.26		1.35	0.56		63	1.57	1.02		1.83	0.94		43	1.22	0.36		1.09	0.29		13	1.07	0.43		1.33	0.65	
**Age**																												
<75	52	3.00	1.35	2.253*	1.40	.57	1.074	28	2.08	1.10	1.466	1.82	.98	.113	23	1.21	0.37	-.108	1.04	0.21	-.761	4	1.30	0.13	1.155	1.75	0.96	1.530
>75	46	2.22	1.10		1.28	.54		35	1.46	0.52		1.79	.91		20	1.22	0.37		1.11	0.30		9	1.00	0.16		1.14	0.38	
**Sex**																												
Male	17	3.43	1.52	-3.387**	1.59	.71	-1.928	10	2.27	1.41	-1.706	2.30	1.25	-1.765	8	1.23	0.31	-.075	1.13	0.35	-.337	0	0	0		0.00	0.00	
Female	81	2.30	1.13		1.30	.51		53	1.51	0.50		1.74	.86		35	1.22	0.39		1.09	0.28		13	1.07	0.43		1.33	0.65	
**Education level**																												
Basic	55	2.45	1.25	.372	1.34	.58	-.393	30	1.40	0.48	-2.062*	1.70	.88	-.863	19	1.27	0.40	.872	1.11	0.32	.761	8	1.15	0.51	.550	1.43	0.79	.405
Advanced	43	2.39	1.28		1.37	.54		33	2.11	1.08		1.91	.99		24	1.20	0.34		1.04	0.32		5	1.00	0.25		1.25	0.50	
**Convivence**																												
Alone	42	2.31	1.31	-1.179	1.38	.62	.521	30	2.11	1.04	1.891	2.00	.98	1.593	22	1.23	0.43	.323	1.09	0.29	.503	7	1.08	0.51	-.130	1.17	0.41	-1.069
Accompanied	56	2.51	1.51		1.32	.51		33	1.56	0.56		1.62	.87		21	1.20	0.28		1.05	0.22		6	1.12	0.34		1.60	0.89	
**Confident**																												
Yes	56	2.50	1.50	.978	1.38	.53	.841	35	2.08	1.08	1.510	1.91	.99	.857	27	1.28	0.40	1.437	1.08	0.27	.172	6	1.52	0.52	.130	1.60	0.89	1.069
No	42	2.32	1.32		1.29	.60		28	1.44	0.49		1.70	.87		16	1.11	0.29		1.07	0.25		7	1.51	0.51		1.17	0.41	

*p< .05; **p<.005

Note: basic education = 8 or fewer years of schooling; advanced education =9 or more years of schooling.

Table S3. Difference between sociodemographic variables and developmental self-care practices.

	Technology							Reflection							New						
		Frequency			Variety				Frequency			Variety				Frequency			Variety		
	N	∑	SD	t	∑	SD	t	N	∑	SD	t	∑	SD	t	N	∑	SD	t	∑	SD	t
Participants	23	1.11	0.48		1.04	0.37		29	0.43	0.32		1.00	0.00	-	45	1.34	0.55		1.33	0.64	
**Age**																					
<75	11	0.58	0.58	-1.368	1.09	0.30	.585	12	0.41	0.22	-.074	1.00	0.00	-	26	1.27	0.49	-1.026	1.27	0.53	-.783
>75	12	1.25	1.01		1.00	0.43		17	0.42	0.40		1.00	0.00		19	1.44	1.03		1.42	0.77	
**Sex**																					
Male	5	0.48	0.40	1.237	1.00	0.00	.239	6	1.10	0.59	-2.596**	1.00	0.00	-	11	1.27	0.31	.661	1.38	0.70	.902
Female	18	1.17	0.49		1.06	0.42		23	0.35	0.16		1.00	0.00		34	1.37	1.01		1.18	0.41	
**Education level**																					
Basic	10	1.06	0.31	-.432	1.10	0.32	.640	17	0.36	0.17	-1.142	1.00	0.00	-	21	1.34	0.55	-.005	1.29	0.72	-.463
Advanced	13	1.15	0.59		1.00	0.41		12	0.50	0.47		1.00	0.00		24	1.35	0.56		1.38	0.58	
**Convivence**																					
Alone	9	1.00	0.36	-.804	1.00	0.50	-.448	13	0.50	0.45	1.196	1.00	0.00	-	18	1.47	1.07	1.229	1.39	0.61	.472
Accompanied	14	1.17	0.53		1.07	0.27		16	0.35	0.14		1.00	0.00		27	1.26	0.46		1.30	0.67	
**Confident**																					
Yes	18	1.13	0.51	.256	1.06	0.42	.293	19	0.31	0.16	-2.645*	1.00	0.00	-	36	1.37	0.58	.562	1.31	0.67	-.578
No	5	1.06	0.39		1.00	0.00		10	1.01	0.45		1.00	0.00		9	1.25	0.45		1.44	0.53	

*p< .05; **p<.005

Note: basic education = 8 or fewer years of schooling; advanced education =9 or more years of schooling.
